# Controllable crystal growth and fast reversible crystallization-to-amorphization in Sb_**2**_Te-TiO_2_ films

**DOI:** 10.1038/srep46279

**Published:** 2017-04-11

**Authors:** Guoxiang Wang, Chao Li, Daotian Shi, Qiuhua Nie, Hui Wang, Xiang Shen, Yegang Lu

**Affiliations:** 1 Laboratory of Infrared Material and Devices, The Research Institute of Advanced Technologies, Ningbo University, Ningbo 315211, China; 2 Key Laboratory of Photoelectric Detection Materials and Devices of Zhejiang Province, Ningbo, 315211, China

## Abstract

The structure evolution and crystallization processes of Sb_2_Te-TiO_2_ films have been investigated. The Sb_2_Te-rich nanocrystals, surrounded by TiO_2_ amorphous phases, are observed in the annealed Sb_2_Te-TiO_2_ composite films. The segregated domains exhibit obvious chalcogenide/TiO_x_ interfaces, which elevate crystallization temperature, impede the grain growth and increase crystalline resistance. Compared with that in conventional Ge_2_Sb_2_Te_5_ film, the shorter time for onset crystallization (25 ns) and amorphization (100 ns) has been achieved in as-deposited (Sb_2_Te)_94.7_(TiO_2_)_5.3_ film under 60 mW laser irradiation. The corresponding recrystallization and re-amorphization can also be realized in the film. From Johnson-Mehl-Avrami (JMA) analysis, it is further found that the one-dimensional grain growth with controlled interface is dominant for the film during the fast phase-change process. Therefore, (Sb_2_Te)_94.7_(TiO_2_)_5.3_ film with improved crystallization mechanism is promising for high-stable and fast-speed memory applications.

Phase-change materials are used extensively in optical or nonvolatile electrical data storage because of their large optical or electrical contrast between crystalline and amorphous phases^1^. Phase-change thin films can be used as the recording and nonlinear mask layers in optical storage^2^. In rewritable phase-change optical disks, the amorphous marks are produced after irradiation by a recording laser pulse. Then, information marks are read out through the apparent optical reflectivity difference between the amorphous marks and crystalline background^3^,^4^. The recorded marks can be erased by heating the media to its crystallization temperature which should be below its melting point^5^,^6^. Phase-change films can also serve as a mask layer for super-resolution optical information devices owing to their nonlinear saturable absorption characteristics, which effectively reduce the optical spot size to below diffraction limit^7^. For non-volatile electrical storage, when high voltage is applied as RESET pulse, the information is erased and the mark becomes amorphous. And the bit is written when SET pulse is applied^8^,^9^.

Conventional Ge_2_Sb_2_Te_5_ (GST) that has been widely used in commercial DVD-RAM^10^ attracts great attentions on the phase-change memory (PCM) applications^11^,^12^. However, its properties including poor thermal stability, slow conversion speed, and high power consumption are limited for further applications. Some novel phase-change materials by constructing amorphous-crystalline nano-composite with a small amount of single element, such as Zn^13^, Mg^14^ or dielectric material HfO_2_^15^ are developed for improving thermal stability and reducing power consumption. Compared with the nucleation-dominated GST alloys, binary growth-dominated Sb-Te alloys exhibit faster crystallization speed but their low crystallization temperature and uncontrollable large grain growth limit the practical applications^16^. In fact, Sb-Te alloys are usually doped with single element V^17^, Zn^18^, and C^19^ *et al*. for the improvement in amorphous phase stability and crystalline resistance. Besides, previous studies reported that the addition of dielectric material SiO_2_^20^ or ZnO^21^ into Sb_2_Te_3_ could effectively increase crystallization temperature and crystalline resistance, thus enhancing the thermal stability and reducing the power consumption.

Obviously, nano-composite phase-change material is a new kind of functional storage materials. Its small size effect and dielectric material package function can refine grain growth and increase crystalline resistance so as to reduce the power consumption of the device. Therefore, it is widely used as phase-change layer in PCM for the replacement of conventional single-structure phase-change materials. Nevertheless, some issues still exist in the nano-composite phase-change materials, including (1) The distribution of nanocrystals, crystal size and specific phase precipitation need to be controlled, (2) The interface microstructure stability and crystallization mechanism need to be investigated and clarified for future development.

In this work, the crystallization and microstructure stability of Sb_2_Te-TiO_2_ nanocomposite layer are studied for the potential memory applications. TiO_x_ is more stable in thermodynamic and less resistive in electric than SiO_x_^22^, which can lower the threshold voltage and power consumption in a memory device based on GST-TiO_x_ phase-change layer^22^. As a favored doping element, it is surprising that there are few reports of doping TiO_2_ into Sb_2_Te for memory applications, and the phase-change kinetics in terms of the fundamental nucleation and growth theories are ambiguous. Therefore, the present work aims at investigating how TiO_2_ doping can affect the phase-change behavior and determine phase-change kinetics of the Sb_2_Te-TiO_2_ films during crystallization process. The results confirm that the TiO_2_ embedment is able to stabilize the amorphous state. Moreover, an optimized composition of (Sb_2_Te)_94.7_(TiO_2_)_5.3_ with one-dimensional growth is determined. A reversible fast phase transformation of as-deposited and melt-quenched (Sb_2_Te)_94.7_(TiO_2_)_5.3_ films upon nanosecond pulsed laser irradiation is demonstrated and the film interface microstructure stability during phase-change process is real-time monitored. All these are of crucial importance for the performance improvement in phase-change materials.

## Results and Discussion

**Figure 1:**
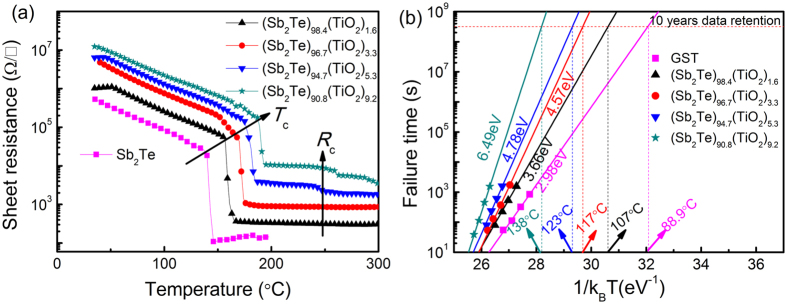
(**a**) Sheet resistance as a function of temperature for undoped and Sb_2_Te-TiO_2_ films; (**d**) The extrapolated data retention time of GST and Sb_2_Te-TiO_2_ films.

Figure 1(a) shows the temperature dependence of the sheet resistance (R-T) upon annealing as-deposited Sb_2_Te-TiO_2_ thin films from ambient temperature to 300 °C with a heating rate of 40 °C min^−1^. According to the R-T behavior, the crystallization temperature (*T*_c_) is determined to be ~156, ~167, ~176, and ~188 °C for the Sb_2_Te films with TiO_2_ content of 1.6, 3.3, 5.3, and 9.2 at%, respectively. These values are significantly higher than that of undoped Sb_2_Te (~144 °C) film. TiO_2_, as the form of chalcogenide/TiO_x_ interfaces, is introduced to Sb_2_Te film, which can inhibit surface atomic motion on nanocrystals^23^ to strengthen the Sb_2_Te-TiO_2_ structure. This accounts for the enhanced *T*_c_ of the Sb_2_Te-TiO_2_ films. Moreover, the crystalline resistance (*R*_c_) is also increased, while amorphous/crystalline resistance ratio remains about 10^4^. The reason is that dispersed TiO_2_ impede the growth and coalescence of Sb_2_Te during recrystallization to form structure discontinuities in Sb_2_Te. The numerous interfacial defects result in the resistance enhancement, indicating that the superheat effect causes the increase in *T*_c_ and finally stabilizes the amorphous Sb_2_Te films.

The data retention is one of the important factors for nonvolatile memories. The maximum temperature for 10-years’ data retention can be extrapolated by fitting the data with the Arrhenius equation: t = τ exp(*E*_a_/*k*_B_T)^24^, where τ, *k*_B_, *E*_a_ are the proportional time constant, Boltzmann’s constant and crystalline activation energy, respectively. The failure time (t) is defined as the time when the sheet resistance reaches half of its initial magnitude at a specific isothermal temperature (T). As shown in Fig. 1(b), the data retention temperatures for 10 years (*T*_10-year_) of the amorphous (Sb_2_Te)_98.4_(TiO_2_)_1.6_, (Sb_2_Te)_96.7_(TiO_2_)_3.3_, (Sb_2_Te)_94.7_(TiO_2_)_5.3_, and (Sb_2_Te)_90.8_(TiO_2_)_9.2_ films are 107 °C, 117 °C, 123 °C and 138 °C with the *E*_a_ of 3.66 eV, 4.57 eV, 4.78 eV and 6.49 eV, respectively. Compared with the conventional GST (88.9 °C, 2.98 eV)^25^, the *E*_a_ and *T*_10-year_ values of TiO_2_-doped Sb_2_Te films are larger. Higher *E*_a_ implies higher energy barrier for crystallization. This is directly associated with improved thermal stability of the amorphous films and prolonged data retention time of memory devices.

**Figure 2:**
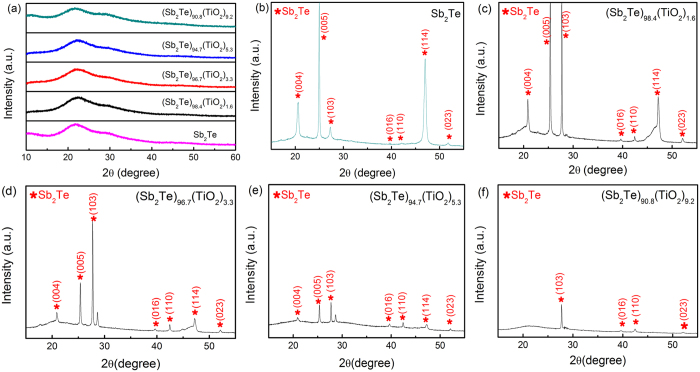
XRD patterns of (**a**) as-deposited Sb_2_Te and Sb_2_Te-TiO_x_ films; 250 °C-annealed (**b**) Sb_2_Te, (**c**) (Sb_2_Te)_98.4_(TiO_2_)_1.6_, (**d**) (Sb_2_Te)_96.7_(TiO_2_)_3.3_, (**e**) (Sb_2_Te)_94.7_(TiO_2_)_5.3_ and (**f**) (Sb_2_Te)_90.8_(TiO_2_)_9.2_.

The XRD was carried out in order to clarify the change in structure. Figure 2(a) shows the XRD patterns of as-deposited Sb_2_Te and Sb_2_Te-TiO_2_ films. No crystallization diffraction peak is observed, indicating that all of the as-deposited films are amorphous. The XRD patterns of Sb_2_Te and Sb_2_Te-TiO_2_ films annealed at 250 °C for 3 min are shown in Fig. 2(b–f). A set of diffraction patterns corresponding to crystalline phase of Sb_2_Te with a hexagonal lattice (*p-*3 ml) appears in Sb_2_Te films as shown in Fig. 2(b). For the films with 1.6 at% TiO_2_ annealed at 250 °C as shown in Fig. 2(c), a change in the preferred orientation of the crystalline Sb_2_Te phase is found that the peak-(103) becomes intense and sharp similar to peak-(005). Such strong peak-(103) becomes dominated as the TiO_2_ content increases to 3.3 at% as shown in Fig. 2(d). Further increase of TiO_2_ content in the Sb_2_Te films can result in a decrease in the intensity of the crystalline peaks as shown in Fig. 2(e), indicating that TiO_2_-doping can suppress the crystalline grain growth. Especially, when TiO_2_ content increases up to 9.2 at% as shown in Fig. 2(f), the peaks (004), (005) and (114) disappear in the (Sb_2_Te)_91.8_(TiO_2_)_9.2_ film. No other phase can be found in all the films annealed at 250 °C. The grain size is calculated based on the full-width at half maximum (FWHM) of (103) preferred orientation using the Scherrer’s equation. It is found that the grain size decreases from ~30 to ~15 nm with increasing TiO_2_ content.

**Figure 3:**
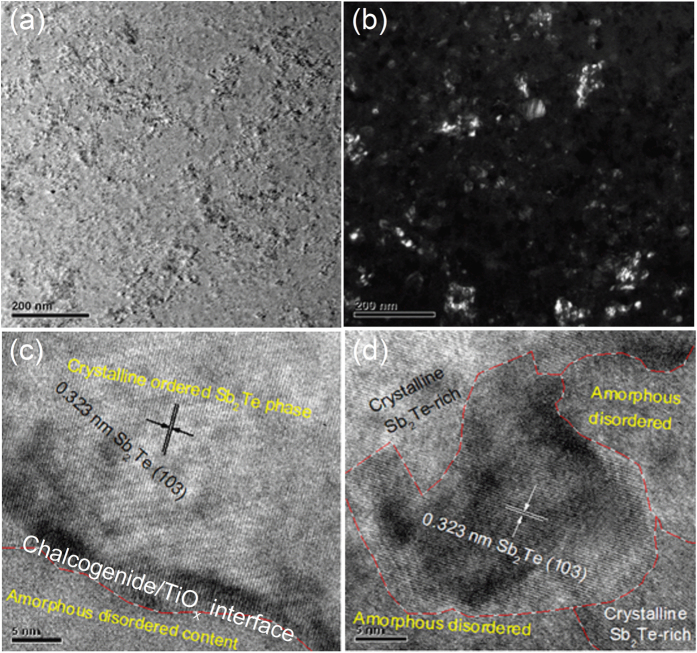
(**a**) The bright-field TEM and (**b**) dark-field TEM images of (Sb_2_Te)_94.7_(TiO_x_)_5.3_ films annealed at 250 °C; (**c**,**d**) high resolution TEM images of the same film.

TEM analysis was performed to examine the microstructural evolution of Sb_2_Te-TiO_2_ films. According to our previous TEM study on pure Sb_2_Te film^19^, when the Sb_2_Te film was annealed at 200 °C, the crystalline Sb_2_Te phase was precipitated with a large average grain size of 20~30 nm. Figure 3(a) shows the bright-field TEM image of Sb_2_Te-TiO_2_ film annealed at 250 °C. The film is crystallized with a small grain size of 10~20 nm. The dark-field TEM image as shown in Fig. 3(b) reveals that the annealed film contains a uniform morphology with embedded dark and bright areas of amorphous contents and crystalline phases. The high-resolution TEM (HRTEM) images as shown in Fig. 3(c,d) makes it possible to measure the interplanar distances and further confirms that the crystal phase is Sb_2_Te. Meanwhile, the (Sb_2_Te)_94.7_(TiO_2_)_5.3_ film contains a certain amount of amorphous contents enriched in TiO_x_ phases around Sb_2_Te crystals, which can interrupt the lattice periodicity, randomize the spatial orientation of Sb_2_Te grains and form Sb_2_Te/TiO_x_ interfaces^26^. This can account for the increase in *T*_c_ and *E*_a_ in the Sb_2_Te-TiO_2_ films. Besides, it also limits sample dimension on the progress of grain growth, and thus affect the crystallization speed^27^.

**Figure 4:**
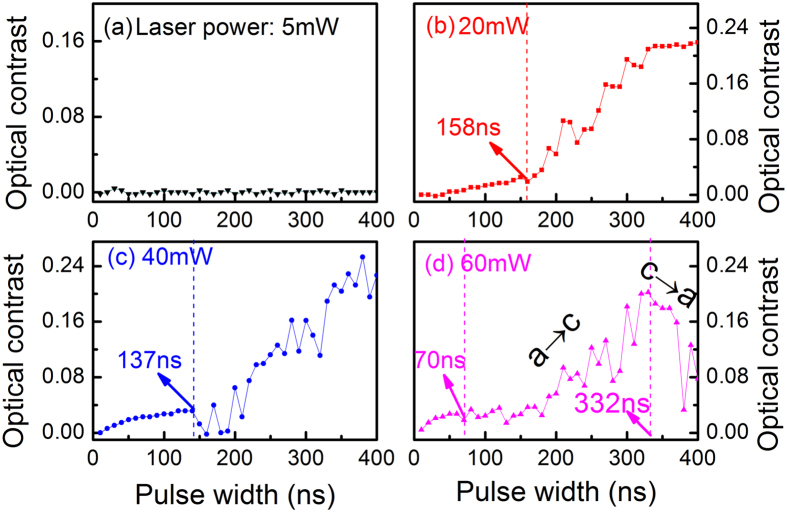
Optical contrast changes of as-deposited amorphous GST films with different laser power and pulse width. (**a**) 5 mW, (**b**) 20 mW, (**c**) 40 mW, (**d**) 60 mW.

The crystallization speed was measured with a static tester under pulsed laser irradiation. The optical contrast ∆*R* of a material is one of the most important optical parameters in phase-change storage^28^, which is defined as ∆*R* = (*R*_c_  − *R*_a_)/*R*_a_^29^, where *R*_c_ and *R*_a_ are the reflectivity of the crystalline and amorphous states, respectively. For the conventional GST, we find that the film exhibits no obvious change in optical contrast after laser irradiation at 5 mW as shown in Fig. 4(a). Under a laser irradiation power of 20 mW, a slight increase in optical contrast is observed as shown in Fig. 4(b) due to the onset crystallization including grain nucleation, forming stable nuclei and subsequent growth until 158 ns. Then a continuous crystallization leads to a gradual increase in optical contrast in the as-irradiated film. With an increasing laser power of 40 mW, the threshold time for crystallization reduces to 137 ns as shown in Fig. 4(c), where the optical contrast gradually increases until 400 ns. Moreover, it is found that the threshold time for crystallization is only 70 ns and a full crystallization can be achieved at 332 ns during the amorphous-to-crystalline (a → c) phase-change process with a laser irradiation power of 60 mW as shown in Fig. 4(d). Subsequently, as the pulse width increases up to 400 ns, the film exhibits an obvious decrease in optical contrast, corresponding to the reverse phase-change process from crystalline to amorphous phases (c → a).

**Figure 5:**
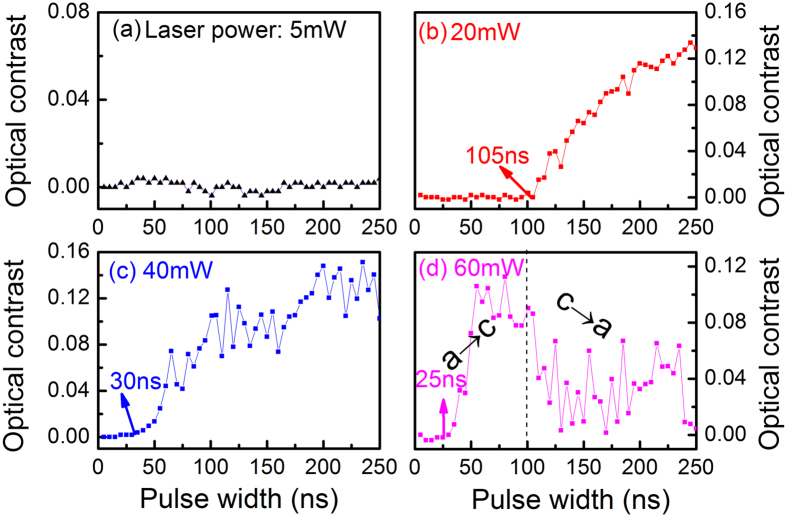
Optical contrast changes of as-deposited amorphous (Sb_2_Te)_94.7_(TiO_2_)_5.3_ films with different laser power and pulse width. (**a**) 5 mW, (**b**) 20 mW, (**c**) 40 mW, (**d**) 60 mW.

In comparison, the optical contrast changes for the as-deposited amorphous (Sb_2_Te)_94.7_(TiO_2_)_5.3_ films irradiated by different laser powers and durations are shown in Fig. 5. When the laser power is 5 mW, there is also no change in the optical contrast as shown in Fig. 5(a) as the pulse width increases from 0 to 250 ns. When the laser power is 20 mW and 40 mW [Fig. 5(b) and (c), respectively], the threshold time for crystallization is determined to be 105 ns and 30 ns, respectively. When the laser power increases to 60 mW, the threshold time for crystallization is around 25 ns and then persist about 100 ns for a complete crystallization as shown in Fig. 5(d). Further irradiation can induce a transition from crystalline to amorphous state. It is due to that the increasing mobility of Sb and Te atoms can be achieved in the film by laser irradiation, and thus the whole system tends to go to an energetically favorable crystalline state^30^. Furthermore, the Sb and Te atoms in the crystalline state migrate from the lattice sites above melting temperature, and these migrated atoms are quenched during the very short laser irradiation process, leading to a partial amorphization of the film. Figure 5(d) demonstrates that both crystallization and amorphization process can be realized in (Sb_2_Te)_94.7_(TiO_25.3_ film at a laser power of 60 mW. Meanwhile, a faster crystallization and amorphization speed can be achieved in the (Sb_2_Te)_94.7_(TiO_x_)_5.3_ films compared with those in the conventional GST.

**Figure 6:**
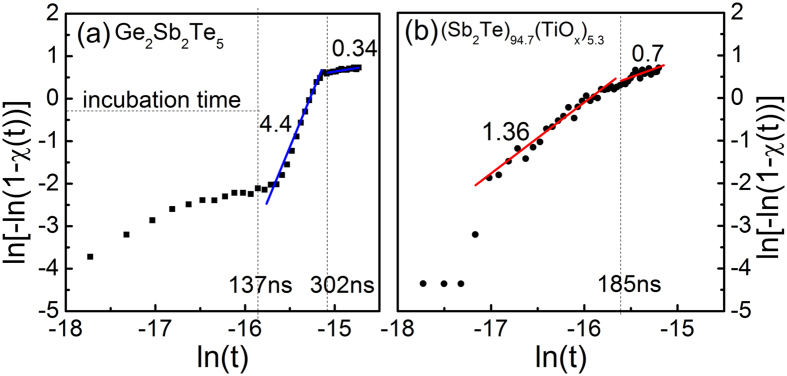
The John-Mehl-Avrami (JMA) plots for the 40 mW laser-induced crystallization of (**a**) GST and (**b**) (Sb_2_Te)_94.7_(TiO_2_)_5.3_ film on the nanosecond time scale.

The John-Mehl-Avrami (JMA) theory^31^ was adopted and JMA plot at laser power of 40 mW was calculated by the JMA model: χ(t) = 1-exp[-(*kt*)^n^], where χ(t), n and *k* is the crystalized fraction of a film, the Avrami coefficient and the effective rate constant, respectively^32^. In this plot, n is the slope of ln(t) versus ln[-ln(1-χ(t))] plot. Although the JMA plot characterizes the isothermal crystallization on the minute time scale, it is also possible to subtract the n from the nanosecond time scale according to previous reports^33^,^34^. As shown in Fig. 6(a), the GST film exhibits an n value of 4.4 in a time scale range from 137 to 302 ns. Up to 137 ns, it is required to generate crystalline nuclei (incubation time). This is in agreement with an n value of 4.8 ± 0.6 in the GST films reported in the previous work^35^, in which grain growth increases with increasing number of nucleation sites. The Avrami coefficient, n, can be separated into two parts, n = a + bc, where a is the nucleation index; b is the dimensionality of the growth such as 1, 2, or 3 for one-, two-, or three-dimensional growth, respectively; and c is the growth index such as 1 or 0.5 for interface or diffusion controlled growth, respectively. After the incubation process as shown in Fig. 6(a), three dimensional growth of the crystalline phase in both the lateral and vertical directions occurs in the laser-irradiated area until the n value reaches 0.3 ns^32^. For GST, the b value can be set to 3 (three-dimensional growth) and c is equal to 1. Then, a is taken to be 1.4, which indicates the number of nuclei per unit volume (N) depending on time as follows, N∝*t*^a^ 35. When pulse width reaches 302 ns, the three-dimensional growth finishes and the n value decreases to 0.34, because crystallization is saturated and limited only to the laser-irradiated area.

The incubation time of the (Sb_2_Te)_94.7_(TiO_2_)_5.3_ is less than 30 ns, which is significantly faster than that of the GST film. It demonstrates that an incubation period is relatively shorter for crystallization. The n value of 1.36 before saturation implies that the crystal growth mechanism is completely different from that of the GST film. From the viewpoint of the crystal growth, the previous equation is also applied to the (Sb_2_Te)_94.7_(TiO_2_)_5.3_ films. The nucleation index a and dimensionality b can be obtained as almost 0.36 and 1, respectively, because the growth is interface controlled growth (c = 1). These values directly indicate the one-dimensional crystalline growth for (Sb_2_Te)_94.7_(TiO_2_)_5.3_ film. As discussed above, the dispersed TiO_2_ provides additional sites and thus promotes heterogeneous crystal growth in (Sb_2_Te)_94.7_(TiO_2_)_5.3_ films. The chalcogenide/TiO_x_ interfaces can accelerate the crystal growth and no more nuclei are generated during the crystal growth (a~0). Therefore, the phase-change speed is extraordinarily fast because the (Sb_2_Te)_94.7_(TiO_2_)_5.3_ films require an extremely short incubation period, which is in good agreement with the results in Fig. 5.

**Figure 7:**
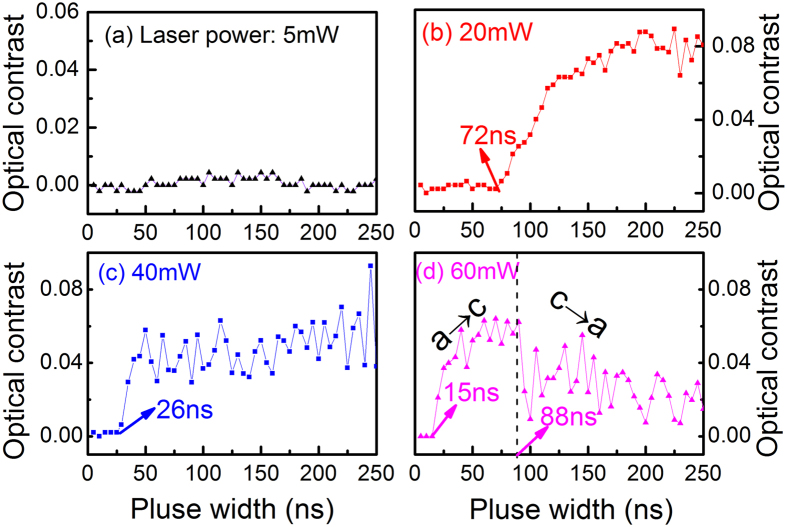
Optical contrast changes of melt-quenched amorphous (Sb_2_Te)_94.7_(TiO_2_)_5.3_ films with different laser power and pulse width. (**a**) 5 mW, (**b**) 20 mW, (**c**) 40 mW, (**d**) 60 mW.

To confirm the microstructure stability between the first and second crystallization effect, the phase-change properties of melt-quenched amorphous films were also measured as shown in Fig. 7. When the laser power is 5 mW [Fig. 7(a)], there is no crystallization as the pulse duration increases from 0 to 250 ns, which is similar to the result in Fig. 5(a). As the laser power increases, the onset recrystallization time of the melt-quenched amorphous film is measured to be 72 ns at 20 mW [Fig. 7(b)], 26 ns at 40 mW [Fig. 7(c)], and 15 ns at 60 mW [Fig. 7(d)], respectively. Moreover, the film scanned by the pulsed laser at a power of 60 mW exhibits a complete crystallization at 88 ns. It is found that the melt-quenched spots are produced in this crystalline area as the longer pulse durations are applied, confirming that re-amorphization of the films can happen and the microstructure stability during a second phase transformation process can be controlled. In addition, the melt-quenched amorphous (Sb_2_Te)_94.7_(TiO_2_)_5.3_ films exhibit much shorter crystallization and amorphization times at the same laser power compared with the as-deposited amorphous films. This is in accordance with the fact that, the melt-quenched film is more ordered than the as-deposited film^36^,^37^. Therefore, it is believed that the controllable crystal growth, fast crystallization, and reversible cycling stability can be demonstrated in the Sb_2_Te-TiO_2_ films with one-dimensional growth mode.

## Conclusions

In summary, nano-composite TiO_2_-Sb_2_Te films have been investigated in terms of phase transition kinetics and microstructures. The increase in crystallization temperature and activation energy of nanocomposite is ascribed to Sb_2_Te grain refinement and hindrance to grain growth resulted from dispersed TiO_2_ and the emergence of chalcogenide/TiO_x_ interfaces. Compared with conventional GST, the as-deposited (Sb_2_Te)_94.7_(TiO_2_)_5.3_ film exhibits a faster crystallization (30 ns) and a shorter onset amorphization (100 ns) under 60 mW. Furthermore, we experimentally confirm the microstructure stability in a second reversible phase transformation process of crystallization and amorphization. JMA theory reveals that the phase transition is prone to be heterogeneous and one-dimensional growth in the crystallization is confirmed for the (Sb_2_Te)_94.7_(TiO_2_)_5.3_ film.

## Methods

### Sample preparation

Nano-composite Sb_2_Te-TiO_2_ films of ~200 nm thickness were deposited on quartz and SiO_2_/Si (100) substrates by a magnetron co-sputtering method using individual TiO_2_ and Sb_2_Te targets. The chamber was evacuated to 2.2 × 10^−4^ Pa before Ar gas was introduced to a pressure of 2.5 × 10^−1^ Pa for the film deposition. The direct current power (*P*_dc_) was fixed at 15 W and applied to a TiO_2_ target of 50 mm diameter. The amount of TiO_2_ in the Sb_2_Te films was adjusted by varying the radio frequency power (*P*_rf_) applied to the Sb_2_Te target of 50 mm diameter. The *P*_rf_ was fixed at 90, 80, 60, and 30 W in order to vary the TiO_2_-doping concentration. Pure Sb_2_Te and GST films with the same thickness were also prepared for comparison.

### Characterizations

The stoichiometry of the as-deposited films was confirmed by X-ray photoelectron spectroscopy. The concentrations of TiO_2_ in the Sb_2_Te films were determined to be around 1.6, 3.3, 5.3, and 9.2 atomic % (at%). The sheet resistance of the as-deposited films was measured *in situ* using a four point probe in a vacuum chamber built in-house, as a function of temperature (non-isothermal) or time (isothermal). The time resolution for the resistance measurements under isothermal annealing is 5 s. The structure of as-deposited and annealed films was examined by X-ray diffraction (XRD), and Transmission electron microscopy (TEM). The diffraction patterns were taken in the 2θ range of 10–60° using Cu Kα radiation with a wavelength of 0.154 nm and performed under Bragg conditions for samples. The acceleration voltage for TEM is 200 kV. A static laser tester (PST-1, Nanostorage Co. Ltd., Korea) with a wavelength of 650 nm and laser spot size of 1 μm was used to characterize the crystallization and amorphization behavior. The laser power and pulses width was set from 5 to 70 mW and from 5 to 250 ns, respectively.

## Additional Information

**How to cite this article**: Wang, G. *et al*. Controllable crystal growth and fast reversible crystallization-to-amorphization in Sb_2_Te-TiO_2_ films. *Sci. Rep.* **7**, 46279; doi: 10.1038/srep46279 (2017).

**Publisher's note:** Springer Nature remains neutral with regard to jurisdictional claims in published maps and institutional affiliations.
